# Evaluation of the knowledge, practices, and attitudes of community pharmacists towards adverse effects of non-steroidal anti-inflammatory drugs (NSAIDs): a cross-sectional study

**DOI:** 10.1186/s40545-023-00641-1

**Published:** 2023-11-01

**Authors:** Hadeer Ehab Barakat, Christine Nazir Aziz, Salwa Selim Ibrahim Abougalambou

**Affiliations:** Department of Clinical Pharmacy and Pharmacy Practice, Ahram Canadian University, Giza, Egypt

**Keywords:** NSAID, Adverse events, Community pharmacists, Knowledge, Practice, Attitudes, Over-the-counter

## Abstract

**Background:**

Nonsteroidal anti-inflammatory drugs (NSAIDs) are the most commonly used over-the-counter medications for the treatment of pain, fever, and inflammation. Gastrointestinal problems and renal complications are the most frequently observed adverse effects associated with NSAID usage. Therefore, this study aims to evaluate the levels of knowledge, attitude, and practice regarding the adverse effects of non-prescription NSAIDs among community pharmacists in Egypt.

**Methods:**

A 4-month cross-sectional survey, including licenced community pharmacists in Egypt, was conducted. The anonymous Google Forms survey was accompanied by a cover letter explaining its purpose. The survey link was sent to 2000 verified community pharmacist email addresses with clear instructions to complete and submit the questionnaire within 3 weeks. Descriptive and inferential statistical analyses were conducted using IBM-SPSS version 26. The means of variables were compared using analysis of variance test. Pearson correlation was employed to assess the level of linear association between the overall knowledge, practice, and attitude scores. *P*-value ≤ 0.05 was considered statistically significant.

**Results:**

Approximately 80% of community pharmacists in Egypt exhibit moderate-to-good knowledge regarding the adverse effects of NSAIDs. Additionally, 60.6% of them demonstrated a positive attitude and 80.9% showed moderate-to-good practice scores towards preventing such adverse effects. The education level was the only demographic factor with significant effects on the NSAIDs-related knowledge, practice, and attitude scores. Community pharmacists primarily rely on internet sources for knowledge updates. Additionally, there was a significant positive linear correlation between knowledge and attitude (*r* = 0.384, *P* < 0.001), knowledge and practice (*r* = 0.178, *P* < 0.001), and between attitude and practice (*r* = 0.311, *P* < 0.001) among the participants.

**Conclusion:**

Community pharmacists have a vital responsibility to perform screenings, assess patient risk elements, and share knowledge to guarantee the appropriate and safe utilisation of NSAIDs. Given that internet sources are presently the most accessible and used sources of information, governmental directions should prioritise the establishment and enhancement of freely accessible drug information sources for community pharmacists. Further research is necessary to assess the effectiveness of counselling and appropriate guidance provided by community pharmacies in promoting safe and proper drug usage.

## Background

Analgesics, such as nonsteroidal anti-inflammatory drugs (NSAIDs) and acetaminophen, are the most common dispensed over-the-counter (OTC) medications for reducing fever, pain, and inflammation [1, 2]. In an Egyptian study, the prevalence of medication abuse was found to have increased to 86.4%; the most abused medicines were analgesics (96.7%) and cough and cold preparations (81.9%), while antibiotic abuse was 53.9% [3].

The most prevalent adverse effects of NSAIDs are gastrointestinal (GI) issues (such as indigestion, bleeding in the upper GI tract, peptic ulcers, and potentially life-threatening perforation in the GI tract). In addition, they can also cause cardiovascular and renal complications [4–6]. However, the general population’s knowledge of the risk factors associated with NSAIDs is limited. These potentially life-threatening side effects of NSAIDs manifest when the recommended dosage is exceeded, when they are used for a prolonged period, or when numerous NSAIDs are taken simultaneously.

In a study conducted over the course of one week involving 1,326 individuals who used ibuprofen in the United States found that 37% of these ibuprofen users also utilised another NSAID concurrently within the same week [7]. Additionally, according to a study examining patients' knowledge of commonly used medications, it was discovered that merely 17.7% of the patients had knowledge of the maximum recommended dosage of ibuprofen, and only 14% were informed of the contraindications associated with its use [8]. Moreover, the results of a survey that examined the consumers’ knowledge of ibuprofen-containing products showed that even though the participants were highly educated and had adequate health literacy levels, less than half of them were aware of the potential side effects that could be caused by NSAIDs [9]. Wilcox et al., (2005) suggested that the reason for inadequate patients’ knowledge about NSAIDs is related to the fact that both prescribers and pharmacists may have provided inadequate safety education [10].

The community pharmacy serves as a safeguard for individuals administering over-the-counter NSAIDs. Typically, the community pharmacist is the initial and readily available healthcare professional to provide complimentary consultations and advice [11]. Thus, they have an obligation as a professional member of the health care team to educate patients and customers about potential side effects and precautions to minimise the risks associated with the use of nonprescription NSAIDs. In cases where patients have absolute contraindications to NSAIDs or face an increased risk of gastrointestinal and renal injuries, community pharmacists should present alternative treatment options supported by scientific evidence [12]. Therefore, pharmacists should effectively screen patients for risk factors that increase the likelihood of adverse effects from NSAIDs use [1, 12].

In general, there is a scarcity of research examining the knowledge, attitudes, and practices of community pharmacists concerning the proper utilisation of over-the-counter NSAIDs [11]. Moreover, there is a lack of established guidelines for community pharmacists to adhere to that ensure the safe dispensing of NSAIDs to consumers.

To the best of our knowledge, this is the first study to evaluate the level of knowledge, attitude, and practice regarding adverse effects of non-prescription NSAIDs among community pharmacists in Egypt and their relationship with the demographic and professional characteristics of the pharmacists based on a 28-item questionnaire that was developed in Qatar [11]. Additionally, the current study assesses the correlations between knowledge, attitude, and practice among the participants.

## Methods

### Study design and participants

A 4-month cross-sectional survey, including community pharmacists, was conducted in the Egyptian community pharmacies between February 2023 and June 2023. The sample was well educated and has good functional health literacy.

### Sample and sampling technique

Using the Raosoft online calculator, a minimum sample size of 367 was determined based on the following criteria: a 5% margin of error, a 95% confidence interval, an estimated population size of 75,165 community pharmacies [13], and an estimated response distribution of 40% [14]. To ensure a representative sample of community pharmacists and achieve a higher response rate, the research team distributed the survey via Google Form to 2000 valid email addresses of licenced community pharmacists.

### Questionnaire structure

The 28-item questionnaire consists of four sections developed and validated in a Qatari study. The first section consists of inquiries about demographic data such as gender, age, nationality, level of education, and years of experience. The second section consists of inquiries about the current knowledge of community pharmacists related to NSAIDs side effects, and the third section consists of inquiries about the current practices of the proper use of NSAIDs. The final section comprises questions that pertain to the perspectives and opinions of community pharmacists in Egypt regarding the utilisation of NSAIDs.

### Data collection

The survey was conducted anonymously, accompanied by a cover letter that clarified the purpose of the questionnaire, utilising Google Forms as the administration platform. The survey link was distributed to 2000 verified email addresses of licenced community pharmacists, with clear instructions for the pharmacists in charge to complete and submit the questionnaire within a three-week timeframe. In cases where the questionnaire was not returned within 2 weeks, reminder emails were sent to individuals who had not responded yet.

### Scoring of the collected data

For each question related to knowledge, a scoring system was utilised where a correct answer was assigned a score of one, while an incorrect answer received a score of zero. Based on the scores achieved by each participant, three categories were established according to their overall knowledge scores using Bloom's cutoff values. Those who scored between 80 and 100% (8 to 10 points) were considered to have "good knowledge", while those who scored between 60 and 79% (6 to 7 points) fell into the category of “moderate knowledge". On the other hand, pharmacists who scored below 60% (0 to 5 points) were labelled as having "poor knowledge".

For the attitude-related questions, the responses were grouped into three categories. The answer of “yes or strongly agree or agree” was assigned a score of 2, while the answer of “I do not know or neutral” was assigned a score of 1, and the answers of “no or disagree or strongly disagree” was assigned a score of 0. Then, categorisation based on Bloom’s cut-off point was performed. Those who scored between 80 and 100% (10 to 12 points) were considered to have "positive attitude", while those who scored between 60 and 79% (8 to 9 points) fell into the category of "neutral attitude", while scoring below 60% (0 to 5 points) were labelled as having "poor attitude".

For the practice-related questions, the responses were grouped into three categories. The answer of frequency “always” was assigned a score of 2, while the answer of frequency “sometimes or usually” was assigned a score of 1, and the answers of frequency “never” was assigned a score of 0. Then, categorisation based on Bloom’s cutoff point was performed. Those who scored between 80 and 100% (7 to 8 points) were considered to have "good practice", while those who scored between 60 and 79% (5 to 6 points) fell into the category of "moderate practice", while scoring below 60% (0 to 4 points) were labelled as having "poor practice".

### Statistical analysis

The collected data from the questionnaires were analysed using Statistical Package for Social Sciences (IBM SPSS Statistics for Windows, version 26.0). Both descriptive and inferential statistical analyses were conducted to determine the mean, frequency, percentage, and standard deviations. The means of continuous and categorical variables were compared, and the associations between them were examined using an Analysis of Variance (ANOVA) test.

The Pearson product-moment correlation coefficient (*r*) was employed to assess the level of linear association between the overall knowledge, practice, and attitude scores. The p-value of 0.05 or less was considered statistically significant in determining the outcome.

### Ethical approval

The Institutional Review Board (IRB) at Ahram Canadian University thoroughly examined and granted approval for the study protocol and questionnaire (ACU-REC1623).

## Results

### Socio-demographic data

Over a span of four months, an online survey was completed by 751 community pharmacists, resulting in a response rate of 37.5% (751 out of 2000) among the targeted population of community pharmacists practising in Egypt. The survey link was shared with them via email. Detailed information regarding the sociodemographic and professional attributes of the participating community pharmacists is shown in Table 1.

**Table 1:** Characteristics pertaining to the demographic data and professional profiles of community pharmacists in Egypt (*n* = 751)

Variable	Frequency (%)
*Gender*	
Male	402 (53.4%)
Female	349 (46.3%)
*Age*	
< 30	512 (68.0%)
30–39	171 (22.7%)
40–50	54 (7.2%)
> 50	14 (1.9%)
*Nationality*	
Egyptian	749 (99.7%)
other	2 (0.3%)
*Years of experience*	
<5 years	485 (64.4%)
5-10 years	181 (24.0%)
> 10 years	85 (11.3%)
*Education*	
Bachelor degree	513 (68.1%)
Master degree	46 (6.1%)
Doctor of pharmacy	112 (14.9%)
other	80 (10.6%)

### Knowledge of renal and gastrointestinal adverse events of NSAIDs among community pharmacists

Table 2 presents the performance of community pharmacists concerning their knowledge towards the renal and gastrointestinal adverse effects associated with NSAIDs. The correct answers are bolded in Table 2.

**Table 2:** Knowledge of community pharmacists on NSAIDs associated renal and GIT adverse effects (*n* = 751)

Knowledge item	Frequency (%)
**NSAIDs are indicated in all of the following conditions except**	
Pain	18 (2.4%)
Acute gout	131 (17.4%)
**Chronic gout**	578 (76.8%)
Headache	24 (3.2%)
**NSAIDs interact with all of the following drugs except**	
Diuretics	184 (24.4%)
Clopidogrel	121 (16.1%)
Warfarin	89 (11.8%)
**Albuterol**	357 (47.4%)
**NSAIDs are contraindicated in all of the following conditions except**	
History of GIT bleeding	63 (8.4%)
Asthma	90 (12.0%)
Hypersensitivity to Ibuprofen	106 (14.1%)
**Osteoporosis**	492 (65.3%)
**Which of the following is a gastrointestinal side effect of NSAIDs**	
**Upper gastrointestinal bleeding**	492 (65.3%)
Inflammatory bowel disease	59 (7.8%)
Irritable bowel syndrome	56 (7.4%)
Crohn’s disease	144 (19.1%)
**Unlike systemic NSAIDs, topical NSAIDs have lower risk of causing epithelial injury in the gastrointestinal tract**	
**True**	633 (84.1%)
False	54 (7.2%)
I do not know	64 (8.5%)
**Inhibition of prostaglandin synthesis causes**	
Decreased gastric acid secretion, increased bicarbonate secretion and increased mucus secretion	133 (17.7%)
**Increased gastric acid secretion, decreased bicarbonate secretion and decreased mucus secretion**	474 (62.9%)
None of the above	144 (19.1%)
**Risk factors for developing gastric injury include all of the following except**	
High dose of NSAID	67 (8.9%)
Previous medical history of gastrointestinal disease (e.g. ulcers)	37 (4.9%)
**Age less than 65 years**	544 (72.2%)
Concomitant steroid therapy of 10 mg/day or greater	103 (13.7%)
**The risk of NSAID-induced kidney injury increases with**	
Lower NSAID doses	40 (5.3%)
**Concomitant use of ACE inhibitors**	565 (75.0%)
Shorter duration of NSAID therapy	76 (10.1%)
Concomitant use of topical capsaicin	70 (9.3%)
**All the following patients should avoid NSAIDs except**	
Patients having a higher risk of stomach bleeding	27 (3.6%)
Patients having stomach problems, including heartburn	53 (7.0%)
Patients having high blood pressure, heart disease, liver cirrhosis, or kidney disease	67 (8.9%)
**Patients with only hypothyroidism, for which they take levothyroxine orally**	550 (73.0%)
Patients taking a diuretic for high blood pressure or heart failure	54 (7.2%)
**Which of the following is the most appropriate alternative for a patient at high risk for NSAID induced acute kidney injury after an acute musculoskeletal injury?**	
Naproxen	88 (11.7%)
Capsaicin cream	134 (17.8%)
**Acetaminophen/paracetamol**	461 (61.2%)
Colchicine	68 (9.0%)

*NSAIDs* nonsteroidal anti-inflammatory drugs

The percentage of correct responses for the knowledge questions varied from 47.4 to 84.1%. The level of knowledge among community pharmacists in Egypt was comparable to that of pharmacists in Qatar. The knowledge question with the highest percentage of correct answers (633/751, 84.1%) was a true or false statement stating that topical NSAIDs have a lower risk of causing epithelial injury in the gastrointestinal tract compared to systemic NSAIDs. On the other hand, the lowest percentage of correct answers (357/751, 47.4%) was related to a question about NSAID drug interactions.

### Practices by community pharmacists to ensure the safe utilisation of NSAIDs to mitigate the risks of renal and gastrointestinal injuries

**Fig. 1:**
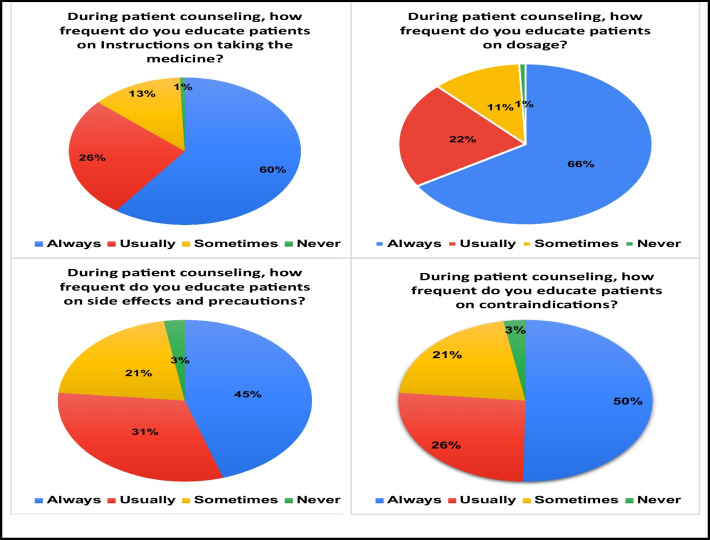
Practice of community pharmacists in Egypt regarding patient education on the safe administration of NSAIDs and the potential adverse effects of NSAIDs (*n* = 751)

Table 3 illustrates that the majority of community pharmacists (43.7%) have updated their knowledge within the past year, followed by 30.1% who updated it between one and five years ago. Among community pharmacists, ibuprofen is the most recommended NSAID. Regarding patient education, a significant percent of community pharmacists (60%) reported always providing instructions on NSAIDs usage, 66% on dosage, 45% on side effects, and 50% on contraindications of NSAIDs, as illustrated in Fig. 1.

**Table 3:** Common practices of community pharmacists in Egypt towards NSAIDs (*n* = 751)

Practice item	Frequency (%)
*When did you last update your knowledge on NSAIDs? (e.g. attended a conference on NSAIDs, read articles on NSAIDs)*	
I did not update my knowledge on NSAIDs	99 (13.1%)
< 1 year ago	329 (43.7%)
1–5 years ago	227 (30.1%)
6–10 years ago	41 (5.4%)
> 10 years ago	18 (2.4%)
I do not recall	37 (4.9%)
*Do you commonly recommend NSAIDs to the customers who visit your community pharmacy?*	
Yes	440 (58.4%)
No	207 (27.5%)
other	103 (13.7%)
*Which NSAID do you usually recommend?*	
Naproxen	52 (6.9%)
Ibuprofen	325 (43.2%)
Diclofenac	219 (29.1%)
Aspirin	84 (11.2%)
Other	71 (9.4%)

*NSAIDs* nonsteroidal anti-inflammatory drugs

**Fig. 2:**
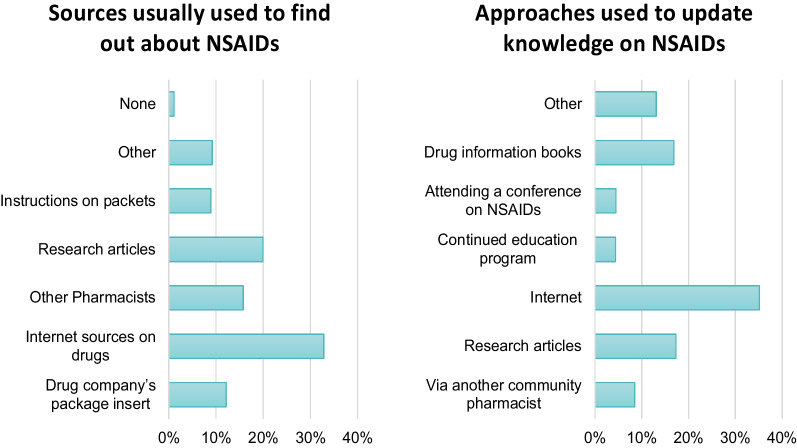
Knowledge sources used by community pharmacists in Egypt (*n* = 751)

According to Table 4, forty-five percent of community pharmacists suggested that switching to a safer drug for gastrointestinal health, such as COX2 selective inhibitors, is the most effective approach to minimising NSAID-related gastrointestinal side effects. Furthermore, 48.1% of community pharmacists believed that switching to a safer drug for kidney health, like acetaminophen, is the best strategy to reduce the renal side effects of NSAIDs. In an inquiry about the least important counselling points regarding NSAIDs, the highest percent of community pharmacists (40.8%) considered it to be the fact that NSAIDs are nonprescription pain relievers in Egypt. Additionally, internet sources are the most commonly utilised resource by community pharmacists (35.1%) to stay updated on NSAID-related knowledge as demonstrated in Fig. 2.

**Table 4:** The opinion of community pharmacists on the best approach to decrease the adverse events of NSAIDs (*n* = 751)

Practice item	Frequency (%)
*In your opinion which of the following measures or approaches are best to use for minimising gastrointestinal side effects of NSAIDs?*	
Reducing the dose of NSAIDs	113 (15.0%)
Changing to a safer drug on the gastrointestinal tract (e.g., COX2 selective inhibitor)	339 (45.0%)
Offering a gastro-protective agent (e.g., antacid, proton pump inhibitors, histamine receptor blockers, misoprostol)	154 (20.5%)
Advising patients to take oral NSAIDs with food or with a full glass of water	145 (19.3%)
*In your opinion which of the following measures or approaches are the best to use for minimising the renal side effects of NSAIDs?*	
Reducing the dose	118 (15.7%)
Changing to a safer drug on the kidney (e.g., acetaminophen)	362 (48.1%)
Advising patients not to use NSAIDs for more than 10 days to relief pain and not more than 3 days to relief fever	219 (29.1%)
Advising patients to take oral NSAIDs with food	52 (6.9%)
*Which of the following is the least important teaching point on NSAID to counsel patients on?*	
NSAIDs are nonprescription pain relievers in Egypt	307 (40.8%)
Before using any medicine ask your doctor/pharmacist if it is safe to use it while using NSAIDs	217 (28.8%)
NSAIDs may not be good in people at risk for kidney disease	154 (20.5%)
Adding NSAIDs to some blood pressure medicines can increase the harm to the kidney	73 (9.7%)

*COX-2* cyclooxygenase-2, *GI* gastrointestinal, *NSAIDs* nonsteroidal anti-inflammatory drugs

### Attitudes of community pharmacists regarding the current approaches on the safe utilisation of NSAIDs to mitigate the risks of renal and gastrointestinal injuries

According to the data presented in Table 5, 70.5% of the community pharmacists are confident that their current knowledge is adequate to provide advice to patients on the safe use of NSAIDs. Additionally, a significant percentage (86.5%) of community pharmacists believe they have a crucial role in preventing adverse events related to NSAIDs. Moreover, a substantial proportion (84.7%) of community pharmacists express their opinion that changes should be implemented in the practice to effectively counsel patients on the appropriate use of NSAIDs, specifically to mitigate the risk of kidney and gastrointestinal side effects.

**Table 5:** Attitude of community pharmacists towards NSAIDs (*n* = 751)

Attitude item	Frequency (%)
*Do you believe that your current knowledge of NSAIDs is sufficient to allow you to advise patients on safe use?*	
Yes	531 (70.5%)
No	100 (13.3%)
I do not know	120 (15.9%)
*Do you believe that community pharmacists have a critical role in preventing NSAIDs related kidney and gastrointestinal injuries?*	
Yes	651 (86.5%)
No	63 (8.4%)
I do not know	37 (4.9%)
*Do you believe that today’s practice in Egypt in relation to counseling on appropriate use of NSAIDs needs to undergo some changes in order to prevent kidney and gastrointestinal side effects?*	
Yes	638 (84.7%)
No	58 (7.7%)
I do not know	55 (7.3%)
*Please indicate your level of agreement with the following statement “It is every patient’s right to be educated on kidney, gastrointestinal and other side effects of NSAIDs.”*	
Strongly agree	382 (50.7%)
Agree	277 (36.8%)
Neutral	80 (10.6%)
Disagree	9 (1.2%)
Strongly disagree	3 (0.4%)
*Please indicate your level of agreement with the following statement “Provision of information on kidney and gastrointestinal side effects of NSAIDs to patients might be time consuming.”*	
Strongly agree	153 (20.3%)
Agree	234 (31.1%)
Neutral	181 (24%)
Disagree	132 (17.5%)
Strongly disagree	51 (6.8%)
*Please indicate your level of agreement with the following statement “In order to avoid dispensing NSAIDs to high-risk patents, pharmacists should ask patients about their health problems and concomitant medication use”*	
Strongly agree	349 (46.3%)
Agree	288 (38.2%)
Neutral	82 (10.9%)
Disagree	23 (3.1%)
Strongly disagree	9 (1.2%)

Using a 5-point Likert scale (strongly agree, agree, neutral, disagree, and strongly disagree), community pharmacists were asked to rate their agreement with five statements. Among the respondents, 87.5% (659 out of 751) agreed (strongly agree and agree) with the statement that it is every patient's right to receive counselling on the kidney, gastrointestinal, and other side effects of NSAIDs. However, a notable percentage of community pharmacists (50.1%, 387 out of 751) expressed strong agreement or agreement with the statement that “providing patients with information about the kidney and gastrointestinal side effects of NSAIDs could be time-consuming".

It was found that 84.5% (637 out of 751) of community pharmacists expressed their agreement with the statement that, in order to prevent dispensing NSAIDs to patients at high risk, it is important for pharmacists to inquire about their health conditions and concurrent medication usage.

**Fig. 3:**
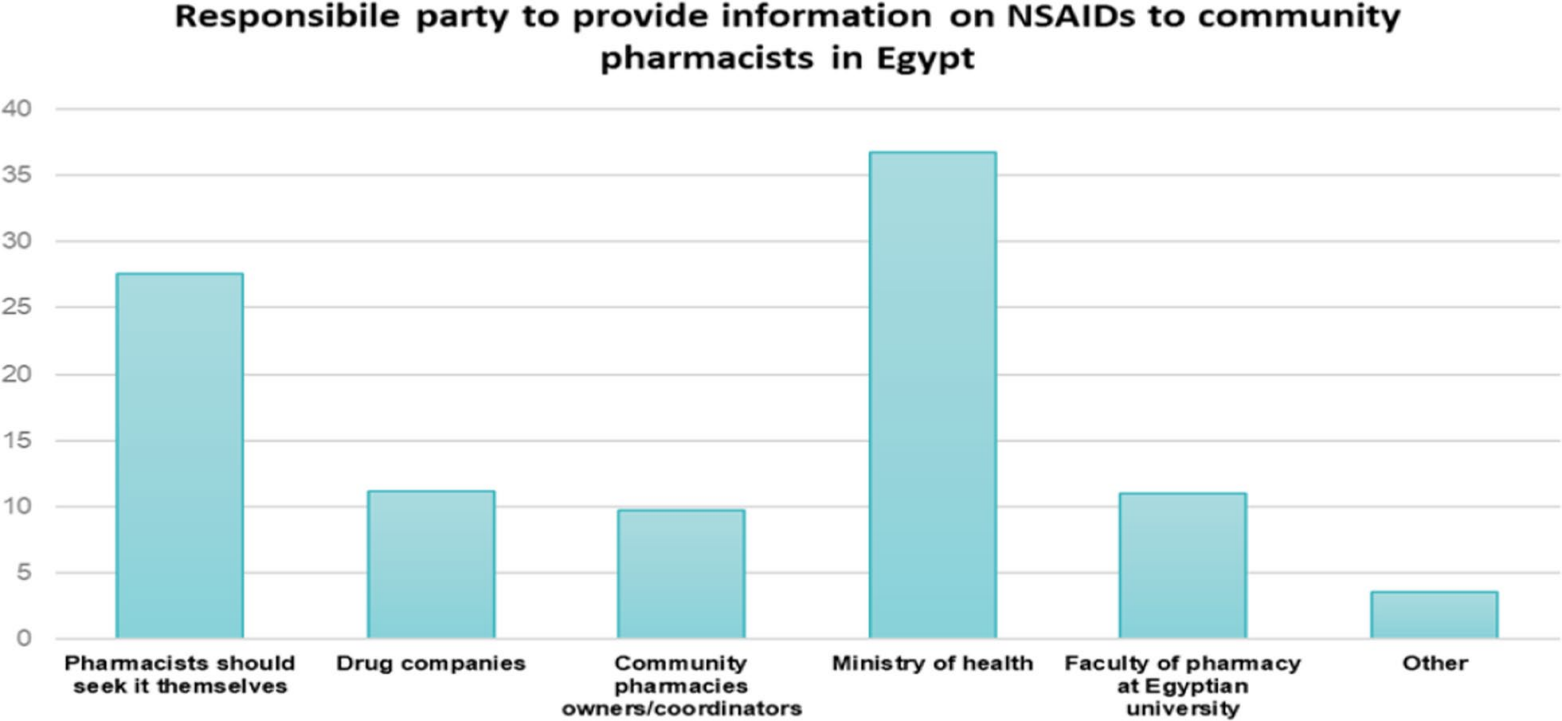
Responsible party to provide information for community pharmacists in Egypt (*n* = 751)

Among the community pharmacists in Egypt, 36.7% believe that the Ministry of Health is responsible for providing information to them, while 26.7% feel that it is the responsibility of the pharmacists themselves to acquire relevant information, as shown in Fig. 3.

### Relation between participants socio-demographic and professional characteristics with knowledge, attitude, and practice scores

Table 6 shows that the education level has significant effects on the NSAIDs adverse effects-related knowledge, practice, and attitude mean scores (*P* < 0.05). On the other hand, there were no significant differences between gender, age, nationality, or years of experience in all scores assessed.

**Table 6:** Relation between mean knowledge, practice, and attitude scores with participants’ socio-demographic and professional characteristics

Variables	Knowledge Mean (± SD)	*P*-value	Practice Mean (± SD)	*P*-value	Attitude Mean (± SD)	*P*-value
*Gender*						
Male	6.78 (1.87)	0.271	6.16 (1.57)	0.850	9.50 (1.85)	0.741
Female	6.93 (1.95)		6.13 (1.55)		9.54 (1.62)	
*Age*						
< 30	6.89 (1.96)	0.153	6.14 (1.57)	0.978	9.53 (1.78)	0.227
30–39	6.89 (1.57)		6.19 (1.51)		9.62 (1.54)	
40–50	6.65 (2.24)		6.13 (1.60)		9.25 (1.98)	
> 50	5.79 (2.20)		6.07 (1.73)		8.78 (1.92)	
*Nationality*						
Egyptian	6.85 (1.91)	0.913	6.15 (1.56)	0.299	9.52 (1.75)	0.984
Other	7 (1.41)		5.0 (1.4)		9.50 (0.71)	
*Years of experience*						
< 5 years	6.77 (1.67)		6.13 (1.57)		9.54 (1.77)	
5–10 years	6.89 (1.97)	0.77	6.12 (1.55)	0.613	9.53 (1.68)	0.708
> 10 years	6.81 (2.00)		6.3 (1.56)		9.37 (1.75)	
*Education*						
Bachelor’s degree	6.96 (1.86)	0.003*	6.13 (1.56)	0.031*	9.68 (1.63)	< 0.001*
Master’s degree	6.13 (2.09)		5.61 (1.37)		8.71 (2.48)	
Doctor of pharmacy	6.48 (2.05)		6.26 (1.63)		9.00 (1.91)	
Other	7.11 (1.73)		6.43 (1.51)		9.69 (1.51)	

*Significant *p* value < 0.05, maximum knowledge score = 10, maximum practice score = 8, maximum attitude score = 12

### Level of NSAIDs-related knowledge, attitude, and practice among community pharmacists

Table 7 presents the distribution of participants' levels of knowledge, attitude, and practice regarding NSAIDs adverse effects. In terms of knowledge scores, with a maximum score of ten, 80.2% of the participants achieved moderate-to-good levels (based on the responses to questions in Table 2). For the attitude scores, with a maximum score of 12, 60.6% of participants demonstrated a positive attitude while 11.6% showed negative attitude (based on the responses to questions in Table 5). Moreover, the practice scores, with a maximum score of 8, 80.9% of participants displayed moderate-to-good practice (based on the responses to the questions presented in Fig. 1).

**Table 7:** The distribution of participants based on their level of NSAIDS-related knowledge, attitude, and practice

Variable	Frequency (%)
*Knowledge scores*	
Good (8–10)	293 (39.0%)
Moderate (6–7)	310 (41.2%)
Poor (0–5)	148 (19.7%)
*Practice scores*	
Good (7–8)	326 (43.4%)
Moderate (5–6)	282 (37.5%)
Poor (0–4)	143 (19.04%)
*Attitude scores*	
Positive	456 (60.6%)
Neutral	208 (27.6%)
Negative	87 (11.6%)

Good/positive, > 80% of the maximum scores; moderate/neutral, 60% to <80% of the maximum scores; poor/negative, <60% of the maximum scores

### Correlations between knowledge, attitude, and practice of NSAIDs adverse events

As shown in Table 8, there was a significant positive linear correlation between knowledge and attitude (*r* = 0.384, *P* < 0.001), knowledge and practice (*r* = 0.178, *P* < 0.001), and between attitude and practice (*r* = 0.311, *P* < 0.001) among the participants.

**Table 8:** Correlation between knowledge, attitude, and practice among community pharmacists

Variables	Pearson correlation coefficient	*P* value
Knowledge and attitude	0.384*	< 0.001
Knowledge and practice	0.178*	< 0.001
Attitude and practice	0.311*	< 0.001

*Significant *P* value < 0.001

## Discussion

Patients consider the community pharmacist a critical healthcare provider who contributes to medication usage and adherence decisions. Patients' health outcomes are influenced by the counselling points provided by community pharmacists during medication dispensing, particularly in terms of medication adherence and the appropriate use of medications [15, 16]. Notably, interactions between pharmacists and patients can have safety benefits beyond the scope of the present findings, particularly in relation to poor adherence to prescribed gastroprotection [17, 18]. Given the frequent interactions between community pharmacists and patients who are prescribed long-term medication, this presents a valuable opportunity to engage and empower patients regarding the safe use of NSAIDs [19].

As community pharmacies play a vital role in the distribution of nonsteroidal anti-inflammatory drugs (NSAIDs), pharmacists should screen potential purchasers and individuals presenting prescriptions to identify any risk factors and provide them with pertinent safety information about these medications [20]. Acute kidney injury and gastrointestinal complications are examples of long-term complications that may be averted by primary interventions administered in community pharmacies concerning NSAIDs [21, 22]. Moreover, these interventions can improve patients knowledge and understanding of safety issues [23].

This cross-sectional, questionnaire-based study was conducted in Egypt to evaluate the knowledge, practices, and attitudes of community pharmacies regarding the adverse gastrointestinal (GI) and renal effects of nonsteroidal anti-inflammatory drugs (NSAIDs).

The implication for practice is that pharmacists should prioritise both efficacy and safety when recommending a treatment. In the current study, it was found that 80.2% of the community pharmacists in Egypt showed moderate-to-good knowledge scores regarding OTC NSAIDs adverse events. The knowledge scores in the current study were less than those of community pharmacists in Qatar, which showed good knowledge in 90% of the pharmacists. However, we should take into consideration that the analysis of good knowledge was based on more rigorous scores than that in the Qatari study [11], in which, good knowledge scores ranged between 8 and 10, and moderate scores ranged between 6 and 8 while those in Qatar good scores ranged between 5 and 10. Community pharmacists in Egypt showed the least knowledge towards drug–drug interaction, although the availability of feasible tools for quick access to check for interactions.

Regarding the practice, more than 80.9% of participants showed moderate-to-good practice scores towards NSAIDs adverse events education, counselling, and identifying high-risk patients who should avoid OTC NSAIDs. The findings in the current study exceed those in the study by Phueanpinit et al., and Babelghaith et al., which revealed that more than 30% of the patients receive insufficient information about potential NSAID adverse effects [12, 18].

The findings in the current study indicate that a considerable number of community pharmacists effectively educated the patients about the proper and safe use of NSAIDs. It was found that more than 60% of the community pharmacists counsel and educate patients about the dosage and instructions for using NSAIDs. In addition, 45% to 50% of the community pharmacists educate the patients about the side effects and contraindications of using NSAIDs.

The highest proportion of pharmacists indicate that they update their knowledge via internet sources, and 70.5% of them believe they have enough knowledge about NSAIDs. Additionally, more than 80% of the pharmacists believe that they have a crucial role in preventing NSAIDS adverse events and that changes are needed in the practices of the pharmacists in Egypt.

Sixty percent of the participants in our study had a relatively high level of positive attitudes towards the adverse effects of NSAIDS. However, although 87.5% of the pharmacists agreed with the patients’ right to be educated about NSAIDs adverse events, more than half of them thought that providing information on NSAIDs side effects to patients might be time-consuming.

Furthermore, a critical incompetency among pharmacists in Egypt was the fact that around 15% of the participants expressed neutral opinion, disagreement, or strong disagreement regarding the attitude item that pharmacists should inquire about patients' health issues and concurrent medication usage to prevent the dispensing of NSAIDs to high-risk individuals. This incompetency may be attributed to various factors, whether pharmacist-related factors, including insufficient knowledge, limited time, inadequate training, lack of motivation, or inadequate compensation [24]. Alternatively, it might also be attributed to patient-related factors, such as patients' disinterest or time constraints. Continuing pharmaceutical education programmes and accreditation assessments for declaring pharmacists suitable for the provision of pharmaceutical care service and renewal of their practice license are crucial approaches that could largely solve this problem [25]. The learning programmes should include consultation skills, history taking, recognition of red flag symptoms indicative of more serious disease and thus warranting referral, clinical observations and assessment skills, safety-netting, and the importance of accurately documenting consultations.

Unlike pharmacists in Qatar, who thought that pharmacists should seek information about NSAIDs by themselves [11], the highest percentage of pharmacists in Egypt believe that the ministry of health is the responsible party for the provision of information to community pharmacists. Thus, it is essential to rectify this perception, and the pharmaceutical organisations and pharmacy faculties in Egypt should embrace educational initiatives tailored for community pharmacists [26]. These programmes should aim to enhance their clinical knowledge and skills, enabling them to deliver comprehensive patient care. These educational programmes should specifically address limitations on information available to pharmacists and gaps in pharmacy practice.

Despite the assurance of high-quality services provided by accredited pharmacies, as guaranteed by the pharmacy council, a study conducted in Thailand revealed that patients may not always receive the comprehensive services they expect, particularly concerning commonly used medications. This lack of comprehensive service has been associated with an increased occurrence of adverse drug reactions [12].

In England, a questionnaire about the knowledge and attitude of community pharmacists to facilitate management of low back pain based on scientific evidence, found that a model for enhancing other facets of supported self-care, particularly for conditions involving chronic pain, could be provided by training pharmacists to be more confident and competent in their ability to offer advice on the management of back pain [27]. Interestingly, in the current study, education level is the only demographic characteristic that influences knowledge, attitude, and practice.

Moreover, there was a significant positive correlation between the knowledge and attitude evaluations of pharmacists (*r* = 0.384, *p* < 0.001). Additionally, there was a statistically significant linear correlation between knowledge and practice (*r* = 0.178, *P* < 0.001), and between attitude and practice (*r* = 0.311, *p* < 0.001) among community pharmacists in Egypt. The findings suggest that acquiring more knowledge will result in enhanced attitudes and practice.

Herein, the involvement of community pharmacists in educating and advising patients can decrease the chances of inappropriate use of NSAIDs and the occurrence of negative effects. However, further research is necessary to assess the effectiveness of counselling and appropriate guidance provided by community pharmacies in promoting safe and proper drug usage.

As internet sources are currently the most readily available and used sources of information, so governmental directions and visions should be focused on the provision and development of freely accessible drug information sources for community pharmacists. Additionally, responsible parties in Egypt should raise the awareness of the community pharmacists to access the available drug interaction checker programmes.

The strength of the current study is that different facets of community pharmacists’ knowledge, attitudes, and practices regarding the adverse effects of NSAIDs were investigated. Moreover, the survey was conducted with a substantial sample size of community pharmacists. Furthermore, the current study supplements earlier studies that focused on the knowledge, attitudes, and practices of community pharmacists concerning the adverse effects of NSAIDs. On the other hand, the limitation in the current study is that we could not differentiate between knowledge, practice, and attitudes among different nationalities. In addition, it was not feasible to differentiate between the knowledge, attitude, and practice sores among different regions in Egypt.

## Conclusion

Community pharmacists are in a position to increase patient awareness of the potential risks associated with NSAIDs. They play a crucial role in conducting screenings, evaluating patient risk factors, and disseminating information to ensure the proper and safe use of these medications.

A significant proportion of community pharmacists demonstrate commendable knowledge scores, and the majority exhibit positive attitudes and practices scores. Education level could have significant effects on the knowledge, attitude, and practice scores. On the other hand, there is no association between age, gender, or years of experience with the levels of knowledge, attitude, and practice. Finally, there is an association between good knowledge scores with good practices and positive attitudes among the community pharmacists.

## Data Availability

The data that support the findings of this study are available from the authors upon reasonable request.

## Abbreviations

COX-2: Cyclooxygenase-2
GI: Gastrointestinal
NSAIDs: Non-steroidal anti-inflammatory drugs
OTC: Over the counter
